# Nicotinamide Mononucleotide Does Not Prevent Cardiac Hypertrophy but Attenuates Cardiac Stress and Remodeling Markers in Mice Exposed to Intermittent Normobaric Hypoxia

**DOI:** 10.3390/nu18203334

**Published:** 2026-10-12

**Authors:** Zhen Wang, Mingxuan Wang, Jingling Guo, Yajing Wang, Shijie Wang, Jing Yuan, Ruifu Yang, Yujing Bi, Yuehua Ke

**Affiliations:** 1 Department of Bacteriology, Capital Institute of Pediatrics, Beijing 100020, China; 2 Capital Institute of Pediatrics, Chinese Academy of Medical Sciences & Peking Union Medical College, Beijing 100020, China; 3 State Key Laboratory of Pathogen and Biosecurity, Academy of Military Medical Sciences, Beijing 100071, China; 4 College of Food Science and Biology, Hebei University of Science and Technology, Shijiazhuang 050091, China

**Keywords:** intermittent normobaric hypoxia, myocardial hypertrophy, gut microbiota, NMN, NAD+, *BNP*, *Myh7*

## Abstract

**Background:** Nicotinamide mononucleotide (NMN) is a naturally occurring, biologically active nucleotide that serves as a crucial NAD+ precursor in various physiological conditions. Although intermittent hypoxia is known to substantially induce various health problems, whether NMN protects against injury induced by intermittent normobaric hypoxia remains unclear. **Methods:** In this study, mice were randomly assigned to groups under normoxia, intermittent normobaric hypoxia, and hypoxia with NMN intervention, and the effects of NMN were evaluated by examining organ indices, hematology, histopathology, and 16S rRNA gene amplicon sequencing. **Results:** Intermittent normobaric hypoxia significantly increased heart weight, cardiac index, right ventricular weight and index, spleen index, serum lactate dehydrogenase activity, and the cardiac expression of *BNP* and *Myh7*, indicating pathological cardiac hypertrophy and activation of a ventricular stress response. It also reduced the beta diversity of the gut microbiota and altered its composition. Although NMN intervention did not significantly affect most organ or hematological indices, except for a further increase in serum LDH activity, it significantly attenuated the hypoxia-induced expression of *BNP* and *Myh7* in the right ventricle, restored the spleen weight and index, and changed the composition of the gut microbiota. These exploratory results indicate that NMN does not prevent intermittent normobaric hypoxia-induced cardiac hypertrophy at the organ level but mitigates cardiac stress markers, ameliorates splenic changes, and modulates the gut microbiota. **Conclusions:** These findings suggest that NMN produced selective molecular and systemic effects under hypoxic exposure rather than an overall protective phenotype and warrant further investigation.

## 1. Introduction

Intermittent hypoxia (IH) is a pathological condition characterized by recurrent episodes of reduced oxygen supply followed by reoxygenation. It is most characteristically associated with the rapid hypoxia–reoxygenation cycles of obstructive sleep apnea syndrome [1,2] and also occurs during sustained high-altitude exposure and repeated travel between low and high altitudes [3]. Chronic IH imposes considerable stress on multiple organ systems, particularly the cardiovascular system, and has been associated with hypertension, cardiac remodeling, metabolic disorders, and cognitive impairment [2,4]. Continuous positive airway pressure remains the first-line therapy for obstructive sleep apnea [5]; however, its long-term compliance is limited, and no specific pharmacological intervention has been established to protect against IH-induced organ injury [6]. Identifying effective and well-tolerated agents that attenuate IH-induced damage is therefore of considerable clinical interest.

Nicotinamide mononucleotide (NMN) is a naturally occurring, biologically active nucleotide that directly converts into nicotinamide adenine dinucleotide (NAD+), a critical molecule for maintaining cellular vitality [7]. NAD+ levels decrease with age and are significantly altered in metabolic diseases, whereas increased NAD+ availability provides various protective benefits, including reducing the risk of cardiovascular and cerebrovascular diseases, protecting the liver and kidney from damage, and delaying aging [8]. Numerous studies indicate that NAD+ metabolism is crucial in cardiovascular diseases such as dyslipidemia, hypertension, myocardial ischemia, and myocardial fibrosis [9]. Supplementation with NMN or nicotinamide riboside can improve cardiac structure and function in models of cardiac hypertrophy or myocardial injury caused by ischemia–reperfusion and aging [10]. However, the protective role of NMN against hypoxia-induced cardiac hypertrophy has not yet been reported.

In addition to its direct effects on NAD+-dependent pathways, NMN has been shown to alter the composition and abundance of the gut microbiota in mice [11,12], and the gut microbiota can in turn influence the metabolism and biosynthesis of NMN [13]. Given the increasingly recognized gut–heart and gut–immune axes in cardiovascular and metabolic diseases [14], it remains unclear whether NMN exerts protective effects against IH-induced cardiac injury and whether such effects are associated with remodeling of the gut microbiota [15,16].

In the present study, we established a mouse model of intermittent normobaric hypoxia and systematically evaluated the effects of NMN intervention on body weight, organ indices, hematological parameters, histopathological changes, cardiac stress marker expression, and the gut microbiota [17]. The primary objective of this study was to determine whether NMN supplementation attenuates intermittent normobaric hypoxia-induced cardiac hypertrophy and the expression of cardiac stress markers; the spleen, hematological parameters, and gut microbiota were examined as exploratory secondary outcomes related to the systemic and immune effects of hypoxia. Our results show that although NMN did not reverse IH-induced cardiac hypertrophy at the organ level, it significantly attenuated the hypoxia-induced expression of the cardiac stress markers *BNP* and *Myh7*, ameliorated splenic enlargement, and moderately altered the composition of the gut microbiota. These findings provide new insight into the pharmacological effects of NMN under hypoxic conditions and may inform strategies for the prevention and treatment of IH-related diseases.

## 2. Materials and Methods

### 2.1. Animals

Young female C57BL/6J mice (6 weeks old, weighing 18 ± 2 g) were purchased from Beijing Vital River Laboratory Animal Technology Co., Ltd. (Beijing, China). Mice were housed in the Experimental Animal Center of the Institute of Microbiology and Epidemiology under controlled conditions: a constant temperature of 23 ± 2 °C, a relative humidity of 35% ± 5%, a 12-h light/dark cycle, and ad libitum access to water and food. All equipment and feed were sterilized at high temperature. All animal procedures were conducted in accordance with ethical guidelines and approved by the Medical Ethics Committee of the Academy of Military Medicine (Ethics Review No.: IACUC-DWZX-2017-006).

### 2.2. Experimental Design and Intermittent Hypoxia Treatment

**Figure 1:**
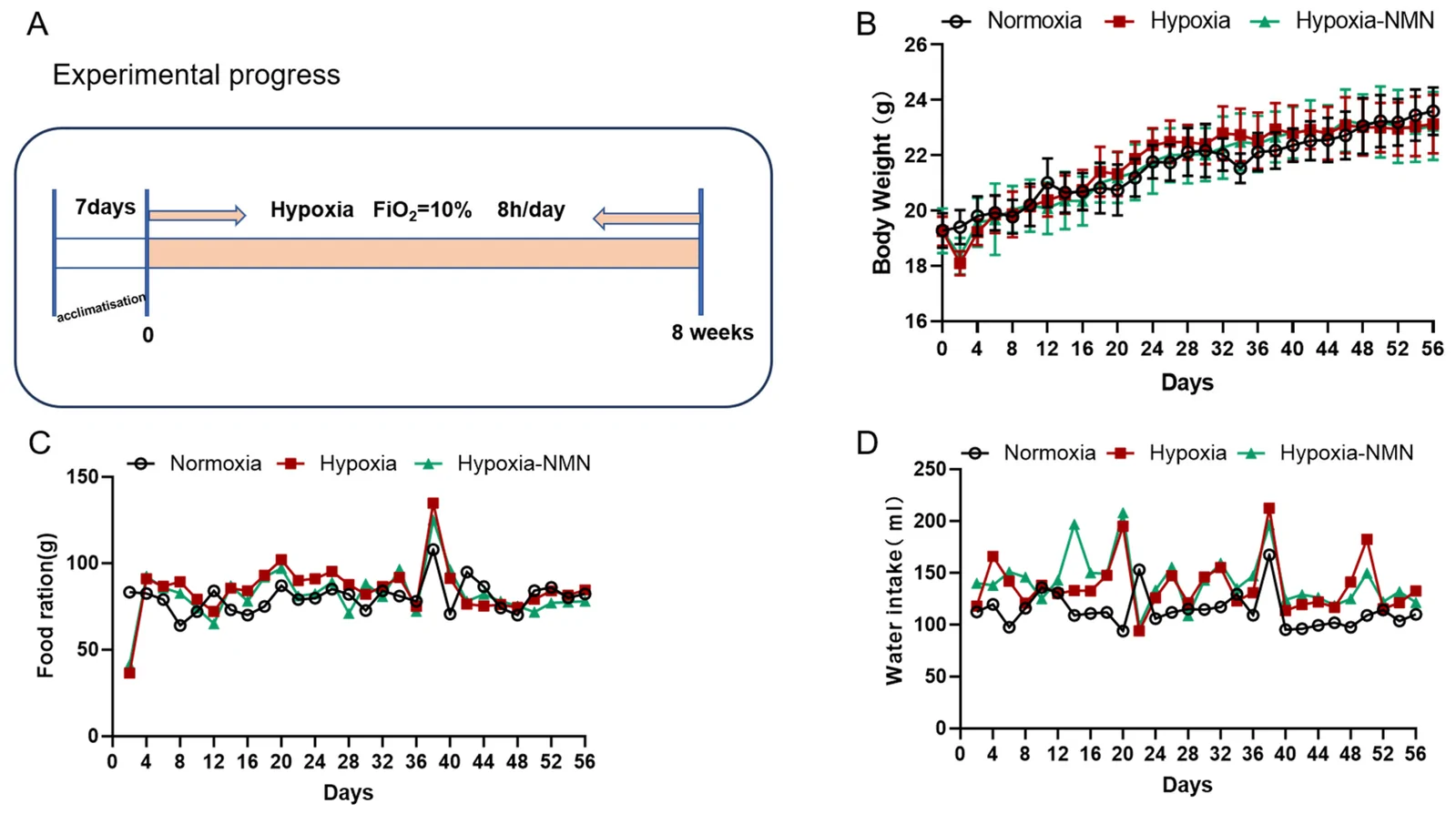
Body weight, food intake, and water consumption of mice exposed to intermittent normobaric hypoxia with or without NMN supplementation. (**A**) Schematic of the experimental protocol. (**B**) Body weight measured every 2 days. (**C**) Food intake per cage measured every 2 days. (**D**) Water intake per cage measured every 2 days.

After a 1-week acclimatization period, mice were randomly assigned to three groups: the normoxia group (*n* = 12), the intermittent normobaric hypoxia group (hypoxia, *n* = 12), and the intermittent normobaric hypoxia with NMN intervention group (hypoxia–NMN, *n* = 12). The normoxia group was housed in the animal center, whereas the other two groups were placed in a hypoxia chamber (Beijing Longfujia Biotechnology Co., Ltd., Beijing, China) in which the oxygen concentration was regulated by a gas mixer supplying N_2_ and compressed air (Figure 1A). The oxygen concentration was set at 10%, with hypoxia applied for 8 h per day, followed by reoxygenation for the remainder of the time; this intermittent normobaric hypoxia was maintained for 8 weeks (56 days) [18]. Because this model involves a sustained daily hypoxic exposure (8 h of continuous 10% O_2_) rather than the rapid hypoxia–reoxygenation cycles characteristic of obstructive sleep apnea, it more closely resembles intermittent high-altitude exposure. NMN was administered ad libitum in drinking water at a dose of 500 mg/kg body weight/day, with the dose adjusted every two days based on average cage body weight and water consumption. Mice were housed 4 per cage (3 cages per group); the cage served as the experimental unit for food intake, water intake, and the actual NMN dose received. Because NMN was delivered via drinking water, the nominal dose of 500 mg/kg/day represents a cage-level average, and the actual individual intake may have varied with water consumption. Because NMN remains stable in water at room temperature for at least 1 week [7], water bottles were replaced every 2 days. Body weight was measured every 2 days, and food and water consumption per cage were recorded every 2 days. Mice were randomly assigned to groups using a computer-generated randomization sequence, and investigators performing organ measurements, histopathological evaluation, and statistical analyses were blinded to group allocation; allocation was concealed from all investigators involved in outcome assessment until the endpoint measurements were completed, and no animals were excluded after randomization. The sample size (*n* = 12 per group) was determined a priori based on preliminary experiments to detect a 15% difference in cardiac index with 80% power at α = 0.05.

### 2.3. Fecal Sample Collection

On days 0 and 56, mice were placed individually in sterile boxes to collect fresh fecal samples; baseline (day 0) samples were stored at −80 °C for possible future analyses, and the day-56 samples were used for the microbiome analyses reported here. The feces were transferred to sterile 1.5-mL centrifuge tubes and immediately stored at −80 °C.

### 2.4. Blood Sample Collection

At the end of the experiment, 200 μL of whole blood was collected into an EDTA-anticoagulated blood collection tube. The number, shape, and proportion of various blood cells were analyzed using a Mindray Veterinary Automatic Blood Cell Analyzer (BC-2800vet, Mindray Bio-Medical Electronics Co., Ltd., Shenzhen, China). The remaining whole blood was transferred to a non-anticoagulated tube, centrifuged at 1000× *g* for 10 min at 4 °C, and the serum was collected for immediate biochemical analysis using a Shenzhen Redu Life Science and Technology Automatic Biochemical Analyzer (Chemray 240, Shenzhen Redu Life Science and Technology Co., Ltd., Shenzhen, China).

### 2.5. Organ Collection

At the experimental endpoint, mice were anesthetized with inhaled isoflurane and euthanized by cervical dislocation prior to tissue harvest. The heart, liver, spleen, and lungs were removed, washed with normal saline, dried with filter paper, weighed, and photographed. The heart was dissected to separate the right ventricle (RV), left ventricle (LV), and interventricular septum, each of which was weighed individually. All tissues were wrapped in aluminum foil, snap-frozen in liquid nitrogen, and stored at −80 °C. The organ index was calculated as the ratio of organ weight (mg) to body weight (g), and the ratio of RV weight to the combined weight of the LV and septum (LV + S) was used to assess right ventricular hypertrophy [19].

### 2.6. Quantitative Real-Time PCR (qRT-PCR) Analysis

RV tissue was ground at low temperature, and total RNA was extracted using the TRIzol method. The concentration and purity of total RNA were measured using a NanoDrop 2000 spectrophotometer (Thermo Fisher Scientific, Wilmington, DE, USA). Two micrograms of total RNA was treated to remove genomic DNA and then reverse-transcribed into cDNA, which was used immediately for qRT-PCR. qRT-PCR was performed using the SYBR Green method on a BIO-RAD CFX Opus 96 Real-Time PCR System (Bio-Rad Laboratories, Hercules, CA, USA) with the following cycling parameters: initial denaturation at 95 °C for 5 min, followed by 40 cycles of denaturation at 95 °C for 10 s and annealing/extension at 60 °C for 30 s. A melting-curve program was used to confirm amplification specificity. Glyceraldehyde-3-phosphate dehydrogenase (*Gapdh*) was used as the internal reference gene; because *Gapdh* is a known HIF-1 target, its Ct values were compared across the three groups and verified to be stable (all *p* > 0.05, one-way ANOVA), and relative expression levels were calculated using the 2^−ΔΔCt^ method. The primer sequences (designed in-house using Primer-BLAST software, National Center for Biotechnology Information (NCBI), Bethesda, MD, USA; online web tool) are listed in Table 1.

### 2.7. Histopathological Analysis

Tissue samples were dehydrated, embedded in paraffin, and sectioned at approximately 4 μm. The paraffin sections were dewaxed, rehydrated, stained with hematoxylin for 3–5 min, washed, differentiated, and blued, followed by eosin staining for 5 min. After dehydration through a graded alcohol series, the sections were sealed and examined under an upright optical microscope (Nikon Eclipse E100, Nikon Corporation, Tokyo, Japan), and images were captured and analyzed using a Nikon DS-U3 camera (Nikon Corporation, Tokyo, Japan).

### 2.8. Fecal Microbiome Analysis

#### 2.8.1. Extraction of Genomic DNA and Amplicon Generation

Total genomic DNA was extracted from the samples using the CTAB method. DNA concentration and purity were monitored on 1% agarose gels, and the DNA was diluted to 1 ng/μL with sterile water. The V3–V4 hypervariable region of the 16S rRNA gene was amplified using the primers 338F (5′-ACTCCTACGGGAGGCAGCAG-3′) and 806R (5′-GGACTACHVGGGTWTCTAAT-3′) with unique barcodes. All PCR reactions were performed with 15 μL of Phusion^®^ High-Fidelity PCR Master Mix (New England Biolabs, Ipswich, MA, USA), 2 μM forward and reverse primers, and approximately 10 ng of template DNA. The thermal cycling conditions were as follows: initial denaturation at 98 °C for 1 min, followed by 30 cycles of denaturation at 98 °C for 10 s, annealing at 50 °C for 30 s, and elongation at 72 °C for 30 s, with a final extension at 72 °C for 5 min.

#### 2.8.2. PCR Product Quantification and Qualification

An equal volume of 1× loading buffer containing SYBR Green was mixed with the PCR products, and electrophoresis was performed on 2% agarose gels for detection. The PCR products were then combined at equal densities, and the mixture was purified using a Qiagen Gel Extraction Kit (Qiagen, Hilden, Germany).

#### 2.8.3. Library Preparation and Sequencing

Sequencing libraries were generated using the TruSeq^®^ DNA PCR-Free Sample Preparation Kit (Illumina, San Diego, CA, USA) according to the manufacturer’s instructions, with index codes added. Library quality was assessed using a Qubit 2.0 Fluorometer (Thermo Scientific, Waltham, MA, USA) and an Agilent Bioanalyzer 2100 system (Agilent Technologies, Santa Clara, CA, USA). Finally, the libraries were sequenced on an Illumina NovaSeq platform, generating 250-bp paired-end reads.

#### 2.8.4. Data Analysis

Paired-end reads were assigned to samples based on their unique barcodes and truncated by removing the barcode and primer sequences. The reads were merged using FLASH, and the resulting sequences were referred to as raw tags. Quality filtering of the raw tags was performed under specific conditions to obtain high-quality clean tags according to the QIIME quality-control process [20]. Sequences were clustered into operational taxonomic units (OTUs) at 97% similarity using UPARSE [21], chimeric sequences were detected and removed using VSEARCH [22], yielding the effective tags for downstream analysis [23]. Taxonomy was assigned against the SILVA database (release 138). Sequencing yielded an average of approximately 60,000 effective tags per sample, and alpha- and beta-diversity analyses were performed on rarefied datasets normalized to the same sequencing depth.

### 2.9. Statistical Analysis

Physiological data are presented as the mean ± standard deviation. Graphs were generated using GraphPad Prism 8.0 software. Because the three groups were independent, differences among them were assessed by one-way ANOVA followed by Tukey’s multiple-comparison test for normally distributed data with homogeneous variances, or by the Kruskal–Wallis test followed by Dunn’s multiple-comparison test when these assumptions were not met; the normality of each variable was verified using the Shapiro–Wilk test and the homogeneity of variances using Levene’s test, and for food intake, water intake, and actual NMN intake, the cage (n = 3 cages per group) served as the experimental unit. Repeated measurements of body weight, food intake, and water intake were analyzed by two-way repeated-measures ANOVA. Genus-level microbiota comparisons were corrected for multiple testing using the Benjamini–Hochberg false discovery rate (FDR). Effect sizes for the principal cardiac outcomes (cardiac index, RV weight, the RV/LV+S ratio, and *BNP* and *Myh7* expression) were quantified as Hedges’ g with 95% confidence intervals (CIs) for the hypoxia vs. normoxia and the hypoxia–NMN vs. hypoxia comparisons. Hedges’ g was calculated from the group means and standard deviations using the small-sample bias-corrected estimator (J = 1 − 3/(4(n1 + n2) − 9)), and 95% CIs were derived from the large-sample standard error of g. Effect sizes were interpreted descriptively (0.2, 0.5, and 0.8 indicating small, medium, and large effects) and were not used for inference, which was based on the ANOVA models described above.

## 3. Results

### 3.1. Effects of NMN on Body Weight, Food Intake, and Water Consumption in Mice Under Intermittent Normobaric Hypoxia

As shown in Figure 1B, exposure to intermittent normobaric hypoxia caused a significant decrease in body weight; however, body weight returned to baseline within 1 week and remained comparable to that of the normoxia group thereafter, indicating that the initial weight loss was transient and that the mice gradually acclimatized to the hypoxic environment. NMN administration did not significantly affect body weight in hypoxic mice at any measured time point.

Food intake was significantly lower in the hypoxia group than in the normoxia group during the first week of exposure, but this difference disappeared from the second week onward (Figure 1C). By contrast, water consumption was significantly higher in the hypoxia group than in the normoxia group throughout the experiment (Figure 1D). NMN intervention did not significantly affect food or water intake.

### 3.2. Effects of NMN on Organs of Mice Under Intermittent Normobaric Hypoxia

#### Effects of NMN on the Heart

**Figure 2:**
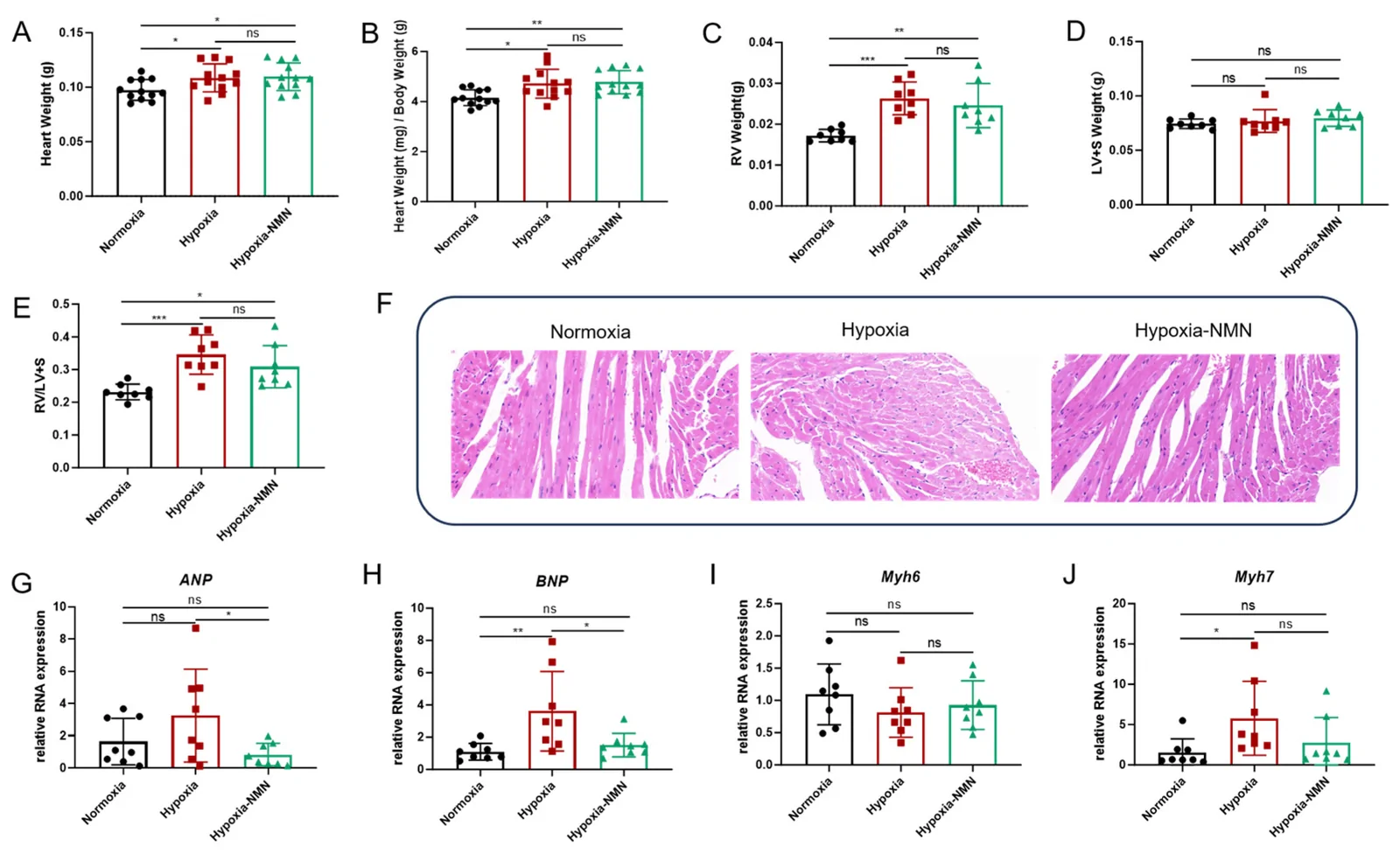
Cardiac hypertrophy and cardiac stress marker expression in right ventricular tissue. (**A**) Heart weight. (**B**) Cardiac index (heart wet weight/body weight). (**C**) Right ventricle (RV) weight. (**D**) Left ventricle plus interventricular septum (LV+S) weight. (**E**) RV index (RV weight/LV+S weight). (**F**) Representative H&E staining of heart tissue (scale bar = 50 µm; original magnification ×400). (**G**–**J**) mRNA expression of hypertrophic markers *ANP* (**G**), *BNP* (**H**), *Myh6* (**I**), and *Myh7* (**J**) in RV tissue, normalized to *Gapdh*. * *p* < 0.05, ** *p* < 0.01, *** *p* < 0.001; ns, not significant (*p* > 0.05).

In our study, mice exposed to intermittent normobaric hypoxia showed significantly increased heart weight, cardiac index, right ventricular (RV) weight, and RV index, indicating marked cardiac hypertrophy (Figure 2A–E). NMN intervention did not significantly alter any of these parameters (Figure 2A–E). The corresponding effect sizes confirmed these observations: intermittent normobaric hypoxia produced large increases in the cardiac index (g = 1.17, 95% CI 0.30 to 2.03), RV weight (g = 2.84, 95% CI 1.45 to 4.23) and the RV/LV+S ratio (g = 2.35, 95% CI 1.08 to 3.63) relative to normoxia, whereas NMN intervention had no appreciable effect on any of these structural parameters (cardiac index, g = 0.11, 95% CI −0.69 to 0.91; RV weight, g = −0.35, 95% CI −1.34 to 0.63; RV/LV+S ratio, g = −0.56, 95% CI −1.56 to 0.44; all 95% CIs included zero).

The number of animals included in each panel is shown by the individual data points (n = 8–12 per group; individual samples were occasionally lost during tissue collection or biochemical assays, and no animals were excluded for any other reason). To further characterize the hypertrophic phenotype at the molecular level, we examined the expression of the hypertrophy-associated markers *ANP*, *BNP*, *Myh6*, and *Myh7* in RV tissue. The mRNA levels of *BNP* and *Myh7* were significantly upregulated by intermittent normobaric hypoxia, whereas those of *ANP* and *Myh6* remained unchanged, indicating activation of a fetal-gene-program-like stress response (Figure 2G–J). Notably, NMN intervention significantly reduced the hypoxia-elevated expression of *BNP* and *Myh7*, suggesting that NMN selectively attenuates the hypoxia-induced ventricular stress response at the molecular level. In terms of effect magnitude, hypoxia produced large increases in both *BNP* (g = 1.34, 95% CI 0.25 to 2.42) and *Myh7* (g = 1.17, 95% CI 0.11 to 2.23) expression relative to normoxia, and NMN intervention produced a large reduction in *BNP* (g = −1.09, 95% CI −2.15 to −0.04) and a moderate reduction in *Myh7* (g = −0.73, 95% CI −1.75 to 0.28) relative to the hypoxia group. Histopathological analysis revealed disordered myocardial architecture and inflammatory cell infiltration in the hypoxia group (Figure 2F). Because these observations were not quantified by blinded semiquantitative scoring or cardiomyocyte cross-sectional area measurement, no conclusion on inter-group differences in tissue morphology can be drawn, and this qualitative description should be regarded as hypothesis-generating only.

### 3.3. Effect of NMN on the Spleen

**Figure 3:**
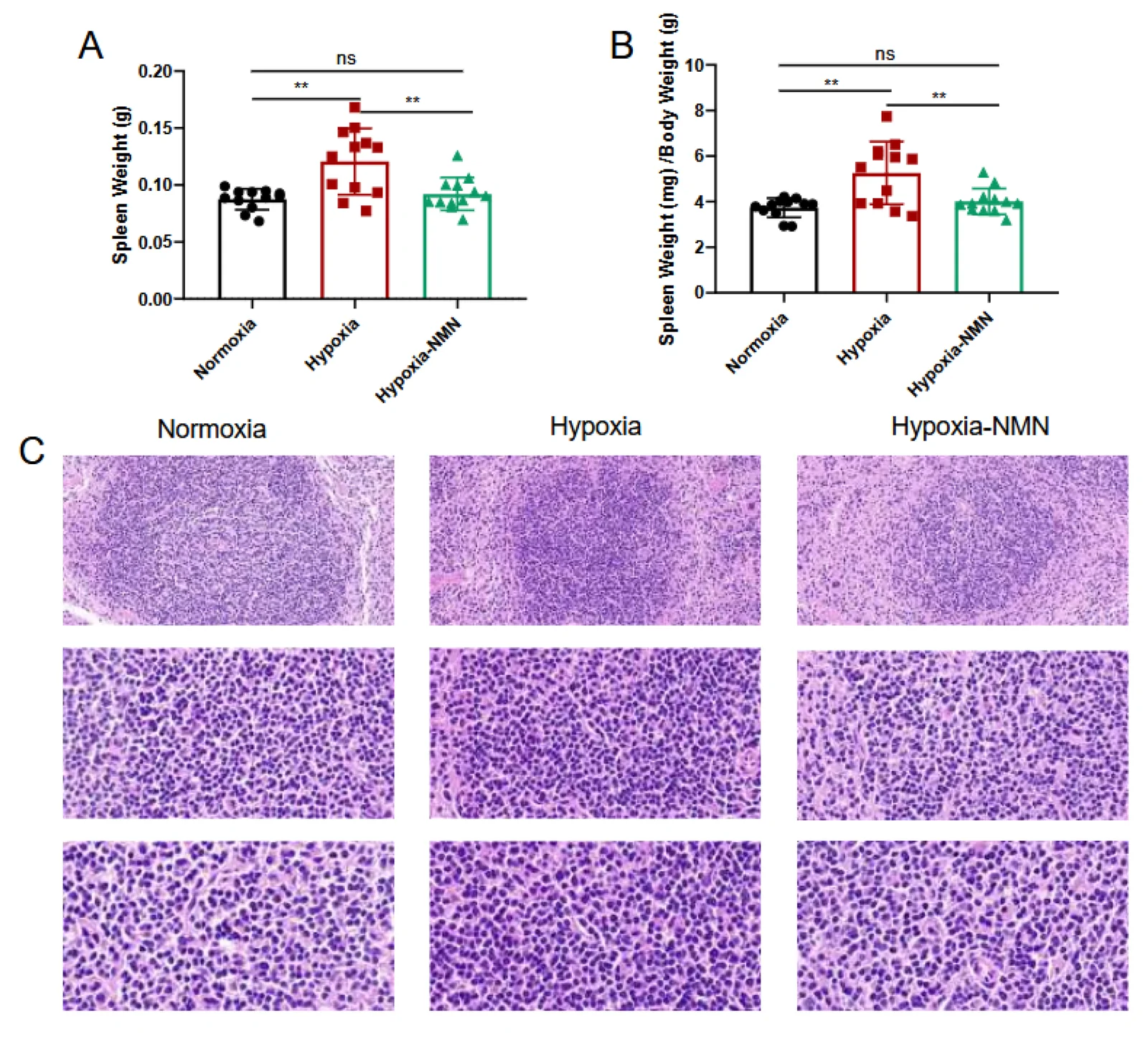
Spleen weight, index, and histology. (**A**) Spleen weight. (**B**) Spleen index (spleen wet weight/body weight). (**C**) Representative H&E staining of spleen tissue (scale bar = 50 µm; original magnification ×400). ** *p* < 0.01; ns, not significant (*p* > 0.05).

The spleen weight and spleen index were significantly increased in the hypoxia group compared with the normoxia group. NMN intervention significantly decreased both parameters, restoring them to levels comparable to those of the normoxia group, which suggests that NMN can ameliorate the effects of intermittent normobaric hypoxia on the spleen [24] (Figure 3A,B). However, H&E staining revealed no obvious histopathological differences among the three groups (Figure 3C).

### 3.4. Effects of NMN on Other Organs

**Figure 4:**
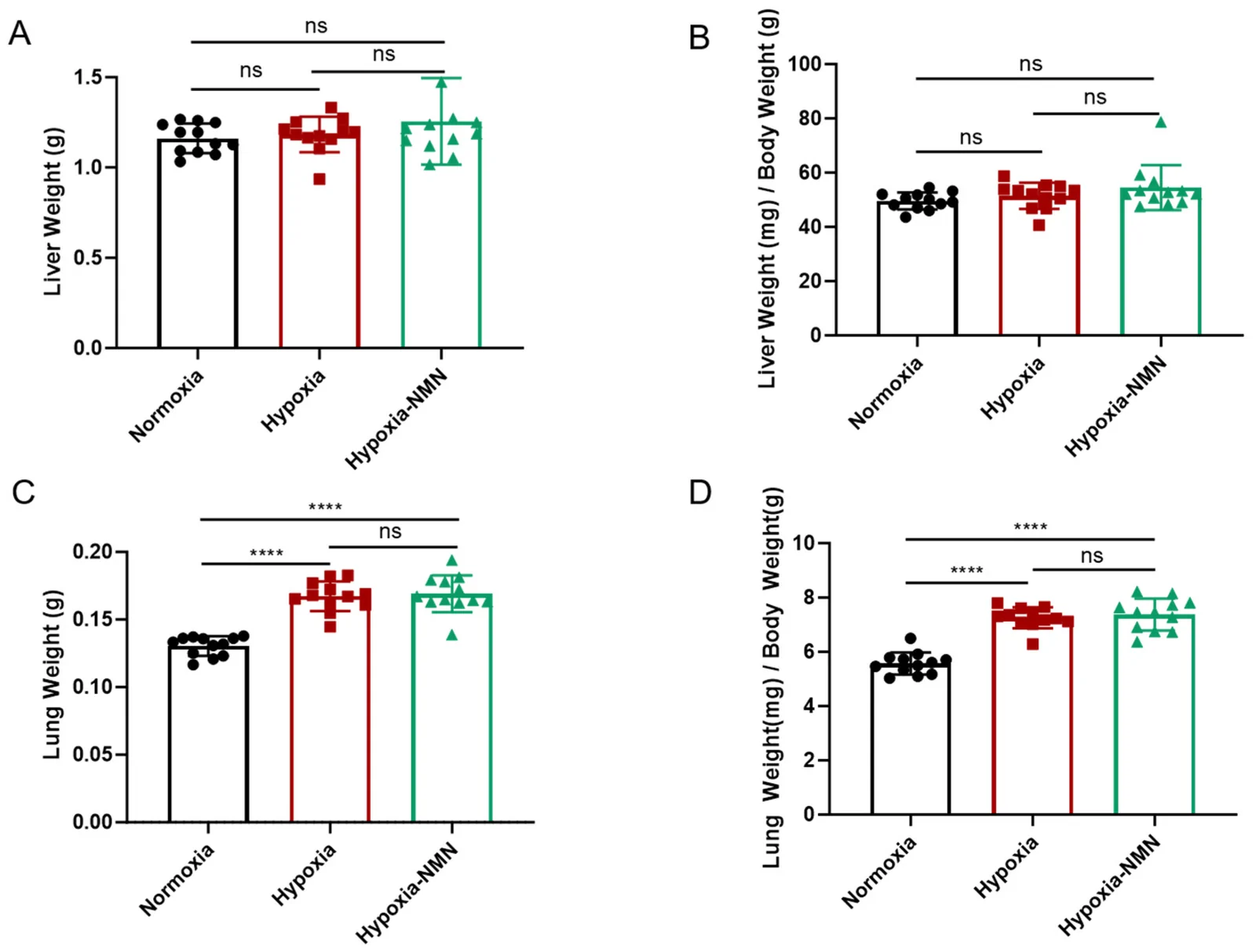
Liver and lung weights and indices. (**A**) Liver weight. (**B**) Liver index (liver wet weight/body weight). (**C**) Lung weight. (**D**) Lung index (lung wet weight/body weight). **** *p* < 0.0001; ns, not significant (*p* > 0.05).

Given that chronic hypoxia can induce pulmonary hypertension, we also evaluated the lungs and liver. After 8 weeks of intermittent normobaric hypoxia, lung weight and lung index were significantly increased, whereas liver weight and liver index remained unchanged (Figure 4A–D). NMN intervention did not affect the weight or index of either organ (Figure 4A–D).

### 3.5. Effects of NMN on Hematological Indexes

**Figure 5:**
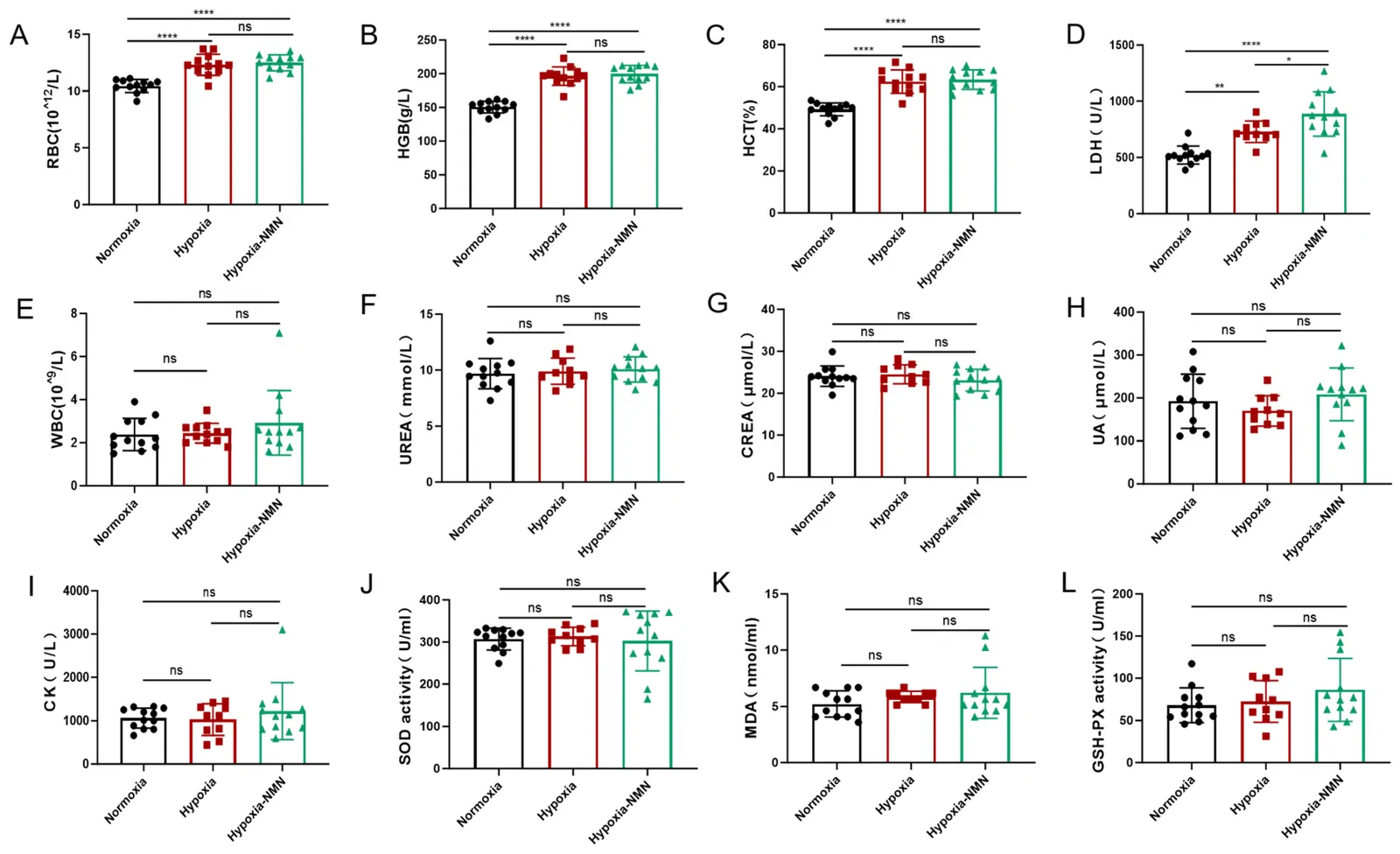
Hematological and biochemical parameters. (**A**) Red blood cell (RBC) count. (**B**) Hemoglobin (HGB). (**C**) Hematocrit (HCT). (**D**) Lactate dehydrogenase (LDH). (**E**) White blood cell (WBC) count. (**F**) Urea. (**G**) Creatinine (CREA). (**H**) Uric acid (UA). (**I**) Creatine kinase (CK). (**J**) Superoxide dismutase (SOD) activity. (**K**) Malondialdehyde (MDA). (**L**) Glutathione peroxidase (GSH-Px) activity. * *p* < 0.05, ** *p* < 0.01, **** *p* < 0.0001; ns, not significant (*p* > 0.05).

Consistent with previous studies, exposure to intermittent normobaric hypoxia significantly increased red blood cell count, hemoglobin concentration, and hematocrit, reflecting a compensatory erythropoietic response to reduced oxygen availability [25,26] (Figure 5A–C). In contrast, white blood cell count, urea, creatinine, and uric acid levels were not significantly altered (Figure 5E–H). Serum lactate dehydrogenase (LDH) activity was significantly elevated in the hypoxia group and further increased in the hypoxia–NMN group (Figure 5D), consistent with hypoxia-induced tissue damage, although LDH is not cardiac-specific, whereas creatine kinase activity, total superoxide dismutase activity, malondialdehyde content, and glutathione peroxidase activity showed no significant changes (Figure 5I–L). NMN intervention did not affect the other hematological or biochemical parameters examined; however, the further increase in serum LDH activity in the hypoxia–NMN group (Figure 5D) indicates that NMN did not prevent hypoxia-induced tissue damage at the biochemical level.

### 3.6. Effects of NMN on Intestinal Microflora

**Figure 6:**
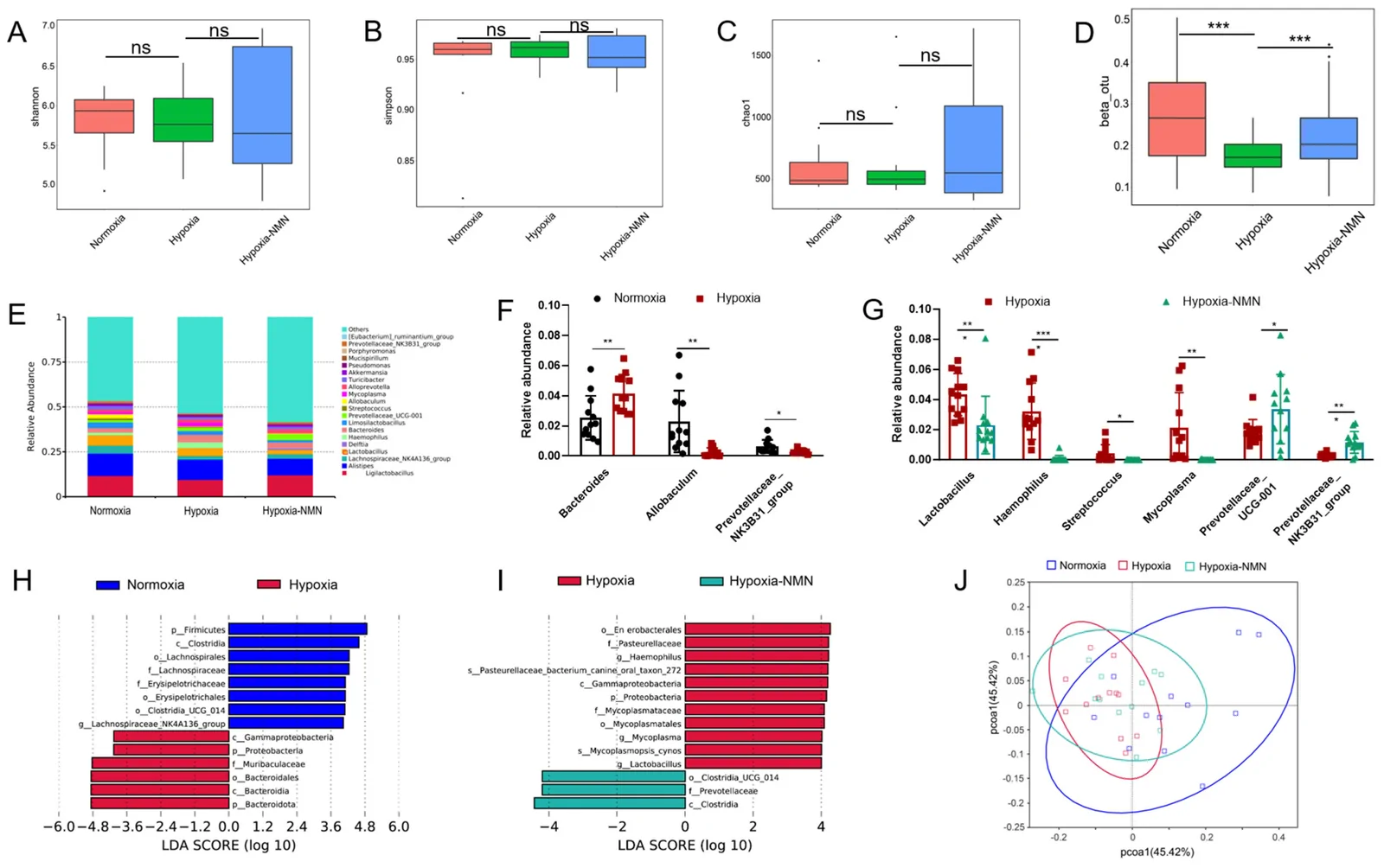
Gut microbiota composition and diversity. (**A**) Shannon index. (**B**) Simpson index. (**C**) Chao1 index. (**D**) Beta diversity index. (**E**) Relative abundance of the top 20 genera. (**F**) Differential abundance of selected genera between the Normoxia and Hypoxia groups. (**G**) Differential abundance of selected genera between the Hypoxia and Hypoxia–NMN groups. (**H**,**I**) LEfSe analysis (LDA score threshold = 4) comparing Normoxia vs. Hypoxia (**H**) and Hypoxia vs. Hypoxia–NMN (**I**). (**J**) Principal coordinate analysis (PCoA) based on Bray–Curtis distance. * *p* < 0.05, ** *p* < 0.01, *** *p* < 0.001; ns, not significant (*p* > 0.05).

Alpha diversity analysis based on the Shannon, Chao1, and Simpson indices revealed no significant differences among the three groups, indicating that neither intermittent normobaric hypoxia nor NMN intervention affected the species richness or evenness of the gut microbiota (Figure 6A–C). In contrast, beta diversity analysis and principal coordinate analysis (PCoA) revealed a significant reduction in the beta diversity index in the hypoxia group and a clear separation of the gut microbial community among the three groups (Figure 6D,J), indicating that hypoxia shifted the composition of the intestinal microbial community and that NMN intervention significantly altered it; PERMANOVA based on Bray–Curtis distances confirmed that the community composition differed significantly among the three groups (*p* < 0.05) [27].

At the genus level, analysis of the top 20 species by relative abundance (Figure 6E) showed that intermittent normobaric hypoxia increased the abundance of *Bacteroides* and decreased the abundances of *Allobaculum* and *Prevotellaceae_NK3B31_group* compared with the normoxia group (Figure 6F). Compared with the hypoxia group, NMN intervention decreased the abundances of *Lactobacillus*, *Haemophilus*, *Streptococcus*, and *Mycoplasma*, and increased the abundances of *Prevotellaceae_UCG_001* and *Prevotellaceae_NK3B31_group* (Figure 6G).

LEfSe analysis further identified taxa with differential abundance between groups; notably, the LEfSe results assigned Haemophilus, Mycoplasma, and Lactobacillus to the hypoxia group. Between the normoxia and hypoxia groups, the normoxia group was enriched in Firmicutes, Clostridia, Lachnospirales, Lachnospiraceae, Erysipelotrichaceae, Erysipelotrichales, Clostridia_UCG_014, and Lachnospiraceae_NK4A136_group, whereas the hypoxia group was enriched in Gammaproteobacteria, Proteobacteria, Muribaculaceae, Bacteroidales, Bacteroidia, and Bacteroidota (Figure 6H). Between the hypoxia and hypoxia–NMN groups, the hypoxia group showed higher abundances of Enterobacterales, Pasteurellaceae, *Haemophilus*, Pasteurellaceae_bacterium, Gammaproteobacteria, Mycoplasmataceae, Mycoplasmatales, *Mycoplasma*, Mycoplasmopsis_cynos, and *Lactobacillus*, whereas the hypoxia–NMN group showed higher abundances of Clostridia_UCG_014, Prevotellaceae, and Clostridia (Figure 6I).

## 4. Discussion

The most notable finding of this study is the dissociation between the structural and molecular cardiac phenotypes. In this study, mice exposed to intermittent normobaric hypoxia for 8 weeks developed pathological cardiac hypertrophy, as evidenced by increased heart weight, cardiac index, RV weight, and RV index, together with upregulation of the cardiac stress markers *BNP* and *Myh7*, elevated serum LDH activity [28], splenic enlargement [24], and marked alterations in gut microbial composition [17]. NMN intervention, however, attenuated the cardiac stress markers and splenic enlargement without reversing the structural hypertrophy, suggesting that the molecular stress response of the myocardium may be more sensitive to NAD+ supplementation than the structural remodeling process itself. Because cardiac function was not assessed and no normoxia-plus-NMN group was included, these exploratory findings should not be interpreted as evidence of cardioprotection.

The most direct indicators of hypoxic stress in the blood include increased red blood cell count, hemoglobin concentration, and blood viscosity [25,26]. Previous studies have shown that red blood cell count and hemoglobin concentration increase in rats and mice following exposure to continuous decompression and hypoxia [24,29]. In this study, after 8 weeks of intermittent normobaric hypoxia, the red blood cell count, hemoglobin concentration, and hematocrit were significantly increased, consistent with previous studies and with the typical hematological response to hypoxic stress. In contrast to the decreased white blood cell count reported in rats with continuous decompression hypoxia [24], no significant change in white blood cell count was observed in our model, which may be attributable to differences in the experimental model and animal species. In addition, Lavie et al. reviewed the oxidative stress responses in patients with obstructive sleep apnea syndrome, including increased lipid peroxidation products such as malondialdehyde [30]; however, no significant changes in antioxidant markers were detected in our mouse model, possibly reflecting differences between chronic intermittent hypoxia in patients and the normobaric hypoxia model. Previous studies have shown that intermittent normobaric hypoxia can damage the heart [6,31]. Accordingly, we observed pathological right ventricular hypertrophy with significantly increased expression of *BNP* and *Myh7*, whereas *ANP* and *Myh6* were unchanged; serum LDH activity was also elevated, further supporting hypoxia-induced ventricular stress response, although LDH is not cardiac-specific. Chronic hypoxia increases pulmonary arterial pressure and cardiac workload [32], which can ultimately lead to right ventricular hypertrophy; in the present study, pulmonary hypertension was assumed on the basis of the sustained hypoxic exposure rather than measured directly by echocardiography or hemodynamics, and this limitation should be considered when interpreting the right ventricular phenotype. In this model, the right ventricular changes most plausibly reflect pressure overload secondary to hypoxia-induced pulmonary hypertension rather than ischemia–reperfusion injury, which is unlikely to occur during sustained exposure to 10% oxygen; accordingly, the attenuation of *BNP* and *Myh7* by NMN may reflect a reduction in ventricular wall stress, possibly mediated in part by improved pulmonary hemodynamics, rather than a direct cardioprotective effect, and these possibilities cannot be distinguished without hemodynamic measurements. The persistently higher water consumption in the hypoxia group may, in addition, reflect enhanced evaporative water loss and altered respiratory patterns under reduced oxygen availability.

The dissociation between organ-level hypertrophy and molecular markers deserves particular attention. Although NMN did not change heart weight, cardiac index, or RV index, it significantly decreased the hypoxia-elevated expression of *BNP* and *Myh7*. *BNP* is a well-established marker of ventricular wall stress, and *Myh7* is a hallmark of pathological cardiac remodeling [33]. The ability of NMN to lower these markers suggests that NAD+ replenishment may attenuate the molecular stress response of the myocardium [34]; however, because myocardial NAD+ was not measured in this study, this mechanistic interpretation is extrapolated from doxorubicin and ischemia–reperfusion models in which NAD+ was measured directly [10,35,36], although this effect was not sufficient to reverse established structural hypertrophy within the 8-week intervention period. Longer treatment duration, higher doses, or earlier intervention may be required to translate this molecular benefit into structural protection.

Intermittent normobaric hypoxia also increased spleen weight and spleen index, which NMN significantly reduced to levels comparable to those of the normoxia group. Splenic enlargement under hypoxia is generally thought to reflect increased erythropoiesis and immune activation; the NMN-induced restoration of spleen size may therefore indicate an immunomodulatory effect of NMN [24]. However, H&E staining revealed no obvious histopathological differences among the groups, and the mechanisms underlying NMN-mediated splenic regulation warrant further investigation.

Hypoxia is closely related to the gut microbiota [14,37]. Ramos-Romero et al. found that intermittent hypobaric hypoxia decreased the proportion of Lactobacilliales and increased that of Bifidobacteriales, with changes in the Firmicutes/Bacteroidetes (F/B) ratio, and reported reduced abundances of Proteobacteria and Clostridia but increased *Bacteroides* [37], consistent with recent observations in mice exposed to chronic intermittent hypoxia [38,39]. In our model, we likewise observed a trend toward increased *Bacteroides* and significantly decreased *Allobaculum* and *Prevotellaceae_NK3B31_group* under intermittent normobaric hypoxia. Regarding alpha diversity, Moreno-Indias et al. reported increased Shannon and Chao1 indices after intermittent hypoxia [27], whereas our model showed no significant change in alpha diversity but a significantly reduced beta diversity index, indicating a compositional shift without a change in species richness. NMN intervention decreased the abundances of *Lactobacillus*, *Haemophilus*, *Streptococcus*, and *Mycoplasma* and increased those of *Prevotellaceae_UCG_001* and *Prevotellaceae_NK3B31_group*, and LEfSe analysis confirmed enrichment of Clostridia_UCG_014, Prevotellaceae, and Clostridia in the NMN-treated group. It should be noted, however, that Haemophilus, Streptococcus, and Mycoplasma are generally regarded as opportunistic pathobionts; although their reduction is consistent with a favorable shift, the functional consequences of these compositional changes require further investigation and should not be over-interpreted. Taken together, these findings demonstrate that NMN can reshape the gut microbial community under hypoxic conditions [11].

NMN plays an important role as an NAD+ precursor. Yamamoto et al. demonstrated that intraperitoneal injection of exogenous NMN protects the heart from ischemia–reperfusion injury [36]. Kuno et al. found that SIRT1 activation counteracts doxorubicin-induced cardiotoxicity in cardiomyocytes [35], and Tang et al. showed that SIRT2 acts as a cardioprotective deacetylase in pathological cardiac hypertrophy [40]. Hsu et al. demonstrated that Nampt, a rate-limiting enzyme of NAD+ salvage, is downregulated during myocardial ischemia–reperfusion and that specific overexpression of Nampt increases myocardial NAD+ levels and provides cardioprotection [41]. These studies support the notion that NAD+ replenishment through NMN can reduce the expression of cardiac stress markers, consistent with the reduced *BNP* and *Myh7* expression observed in our NMN-treated mice [9]. Consistent with this interpretation, NAD+ metabolism has been shown to decline in pathological cardiac remodeling, and NAD+ precursor supplementation ameliorates cardiac hypertrophy phenotypes in mouse models [42,43].

With respect to the gut microbiota, Ru et al. found that NMN supplementation protected intestinal function and modulated the gut microbiota in aging mice [12]. Huang et al. reported that feeding mice different concentrations of NMN increased the abundance of butyrate-producing bacteria (*Ruminococcaceae_UCG_014* and *Prevotellaceae_NK3B31_group*) and probiotics (*Akkermansia muciniphila*) while decreasing the abundance of harmful bacteria (*Bilophila* and *Oscillibacter*) [11]. Consistent with these observations, our study found that NMN intervention in the intermittent normobaric hypoxia model increased the abundance of Clostridia_UCG_014, Prevotellaceae, and Clostridia, supporting a microbiota-modulating role of NMN under hypoxic stress.

Several limitations of this study should be acknowledged. First, only young female mice were used (to reduce within-cage aggression and inter-individual variability), and sex-, age-, and dose-specific effects cannot be excluded, so the present findings should be generalized only to this specific setting. Second, NMN was administered at a single dose (500 mg/kg/day) via drinking water, and neither plasma nor tissue NAD+ levels were measured. Statements regarding NAD+ replenishment are therefore extrapolated from other models, and myocardial NAD+ should be measured directly in future studies if tissue remains available. Third, the absence of a normoxia-plus-NMN group means that the effects of NMN on the spleen, gut microbiota, and gene expression cannot be separated from rescue of a hypoxic phenotype. Fourth, cardiac function was not assessed by echocardiography or hemodynamics, and histological findings were not quantified; terms such as “cardioprotection” and “pathological hypertrophy” are therefore used descriptively and should be interpreted cautiously. Fifth, the causal relationship between gut microbial remodeling and cardiac outcomes was not established. Sixth, although fecal samples were collected at baseline (day 0) and on day 56, only the day-56 samples were included in the present analysis; within-animal longitudinal comparisons and baseline-adjusted analyses that account for potential cage effects were not performed and will be prioritized in future work. Seventh, the effect sizes reported for the principal cardiac outcomes are based on small samples (n = 8–12 per group), and their 95% confidence intervals are correspondingly wide, particularly for *BNP* and *Myh7*, whose between-animal variability was high; these effect estimates should therefore be interpreted as exploratory rather than precise. Nevertheless, these exploratory findings indicate that NMN is associated with attenuation of intermittent normobaric hypoxia-induced cardiac stress marker expression and with modulation of the gut microbiota; whether this translates into clinical benefit for hypoxia-related diseases requires further study. Future studies with multiple doses, NAD+ measurements, and microbiota manipulation experiments are warranted.

## 5. Conclusions

Intermittent normobaric hypoxia induced a cardiac hypertrophic phenotype in mice, increased serum lactate dehydrogenase activity and the expression of the cardiac stress markers *BNP* and *Myh7* and altered the beta diversity and composition of the gut microbiota [17]. NMN intervention did not reverse the organ-level hypertrophy but significantly reduced the hypoxia-elevated expression of *BNP* and *Myh7*, attenuated splenic enlargement, and modulated gut microbial composition, although the further elevation of serum LDH activity and the limited functional characterization of the gut microbial changes caution against a simple protective interpretation. As an exploratory observation, these findings indicate that NMN produced selective molecular and systemic effects under intermittent normobaric hypoxia rather than an overall protective phenotype, and their physiological significance requires further investigation.

## Figures and Tables

**Table 1:** Primer sequences for qRT-PCR for the target genes in the right ventricular tissues in mice.

Gene	Sequence (5′→3′)
*ANP*	Forward: GCTTCCAGGCCATATTGGAG
	Reverse: GGGGGCATGACCTCATCTT
*BNP*	Forward: AGTCCTTCGGTCTCAAGGCA
	Reverse: CCGATCCGGTCTATCTTGTGC
*Myh6*	Forward: TGCACTACGGAAACATGAAGTT
	Reverse: CGATGGAATAGTACACTTGCTGT
*Myh7*	Forward: CCTGCGGAAGTCTGAGAAGG
	Reverse: CTCGGGACACGATCTTGGC
*Gapdh*	Forward: GGTTGTCTCCTGCGACTTCA
	Reverse: TGGTCCAGGGTTTCTTACTCC

## Data Availability

The datasets generated and/or analyzed during the current study are available from the corresponding author upon reasonable request. The raw 16S rRNA gene sequencing data generated in this study have been deposited in the European Nucleotide Archive (ENA) at EMBL-EBI under study accession PRJEB127278.
